# Comparative Trace Elemental Analysis in Cancerous and Noncancerous Human Tissues Using PIXE

**DOI:** 10.1155/2013/192026

**Published:** 2013-05-16

**Authors:** Stephen Juma Mulware

**Affiliations:** Ion Beam Modification and Analysis Laboratory, Physics Department, University of North Texas, 1155 Union Circle, No. 311427, Denton, TX 76203, USA

## Abstract

The effect of high or low levels of trace metals in human tissues has been studied widely. There have been detectable significant variations in the concentrations of trace metals in normal and cancerous tissues suggesting that these variations could be a causative factor to various cancers. Even though essential trace metals play an important role such as stabilizers, enzyme cofactors, elements of structure, and essential elements for normal hormonal functions, their imbalanced toxic effects contribute to the rate of the reactive oxygen species (ROS) and formation of complexities in the body cells which may lead to DNA damage. The induction of oxidative-induced DNA damage by ROS may lead to isolated base lesions or single-strand breaks, complex lesions like double-strand breaks, and some oxidative generated clustered DNA lesions (OCDLs) which are linked to cell apoptosis and mutagenesis. The difference in published works on the level of variations of trace metals in different cancer tissues can be attributed to the accuracy of the analytical techniques, sample preparation methods, and inability of taking uniform samples from the affected tissues. This paper reviews comparative trace elemental concentrations of cancerous and noncancerous tissues using PIXE that has been reported in the published literature.

## 1. Introduction

Studies have shown that the imbalance in the composition of trace metals which are generally recognized to be essential to normal human homeostasis besides accumulation of potentially toxic and nonessential trace metals may cause disease. The significance of the essential trace metals is indisputable due to their positive roles when in specific concentration ranges while on the other hand displaying toxic effects in relatively high or low concentration levels. Physiochemical properties of trace metals govern their uptake, intracellular distribution, and the binding of the metal compounds in biological systems. In spite of diverse physiochemical properties of metals compounds, there are three main predominant mechanisms related to metal genotoxicity. The first is the interference with cellular redox regulation and production of oxidative stress which does cause oxidative DNA damage or trigger signaling cascades that may lead to stimulation of malignant growth [1]. Second is the ability to inhibit the major DNA repairs mechanisms which may result in genomic instability and accumulation of critical mutations. And third is the deregulation of cell proliferation by induction of signaling pathways or ability to inactivate the growth controls such as tumor suppressor genes [2]. 

The essential trace metals have four main functions which include (i) stabilizers, (ii) elements of structure, (iii) essential elements for hormonal function, and (iv) cofactors in enzymes. Inadequate or lack of trace elements will affect the structure alone or will affect structural function due to lack of stabilization, change of charge properties, and allosteric configuration [3]. The deficiency of trace elements as enzyme cofactors is expected to expose the individual to carcinogenic stress [4]. Trace metals form core structures of superoxide dismutase (SOD) which are a group of metalloenzymes (containing Fe, Mn, or Cu and Zn) that catalyze the disproportionate of superoxide free radical (O_2_^•−^) to form hydrogen peroxide and dioxide, hence breaking down the toxic reactive oxygen species (ROS) radical. These enzymes have been considered as the defense system against the cytotoxic superoxide free radical. Cu, Zn-SOD which is a prototypical dinuclear metalloproteinase is an important antioxidant enzyme for cellular protection from ROS and several proteins involved in DNA repair [5]. Metallothionein (MT) is a family of cysteine-rich, low-molecular-weight (MW ranging from 500 to 14000 Da) proteins which have the capacity to bind both physiological (such as zinc, copper, and selenium) and xenobiotic (such as cadmium, mercury, silver, and arsenic) heavy metals through the thiol group of its cysteine residues. Cysteine residues from MTs can capture harmful oxidant radicals like the superoxide and hydroxyl radicals, hence there important role in controlling oxidative stress that would lead to cell mutations. Due to MT's important role in transcription factor regulation, problems with MT function or expression may lead to malignant transformation of cells and even cancer. Research has found increased expression of MTs in some cancers of the breast, colon, kidney, liver, skin (melanoma), lung, nasopharynx, ovary, prostate, mouth, salivary gland, testes, thyroid, and urinary bladder; it has also found lower levels of MT expression in liver adenocarcinoma and hepatocellular carcinoma [4].

More than other 30 enzymes including ceruloplasmin, cytochrome oxidase, lysine oxidase, dopamine-hydroxylase, ascorbate oxidase, and tyrosinase among others in the body have copper as main building block. Some of these enzymes are involved in the synthesis of the main component of connective tissues called collagen [4]. On the other hand, iron plays a significant role in oxygen transportation, xenobiotic metabolism, and oxidative phosphorylation and being a prosthetic group in many enzymes; however, its excessive content in organism's binding capacity can be toxic even leading to cancer development. Most trace metals which occur in various oxidation states including Fe, Cu, Co, Cr, Hg, and V can generate ROS, a property which explains their carcinogenic effects [6–8].

Carcinogenesis is considered to occur in four stages: initiation, promotion, progression, and metastasis. The mechanism of metal-induced carcinogenesis is believed to be involved in all stages of cancer development. Consequently, the roles of trace metals in cancer development and inhibition have a complex character and have raised many questions because of their essential and toxic effects on people's health. In the last few decades, metals such as cadmium, nickel, arsenic, beryllium, and chromium (VI) have been recognized as human or animal carcinogens by the International Agency for Research on Cancer [9, 10]. The carcinogenic capability of these metals depends mainly on factors such as oxidation states and chemical structures. The oxidative concept in metal carcinogenesis proposes that complexes formed by these metals, in vivo, in the vicinity of DNA, catalyze redox reactions, which in turn oxidize DNA. The most significant effect of reactive oxygen species (ROS) in the carcinogenesis progression is DNA damage, which results in isolated base lesions or single-strand breaks, complex lesions like double strand breaks as well as some oxidatively generated clustered DNA lesions (OCDLs) and the sister chromatid exchange [11, 12]. It has been estimated that approximately 2 × 10^4^ DNA damaging events occur in every cell per day; a major portion of these occur via reactive oxygen species (ROS).

The oxidatively induced DNA damage associated with ROS is apurinic or apyrimidinic (abasic) DNA sites, oxidized purines and pyrimidines, and single- and double-strand DNA breaks. Even though the lesions are not ultimately lethal to the cell, they are considered highly mutagenic. The creation of an altered base or base loss is not expected to significantly destabilize the DNA molecule; however, a localized perturbation of the stacking forces, hydrogen bonds, and interaction with water molecules and positive metal ions like Na+ surrounding the DNA double helix is expected to occur [13, 14]. These perturbations generally change the DNA at the lesion site. Peroxyl radicals in the presence of oxygen are also believed to cause lipid peroxidation DNA damage and carcinogenesis [15]. Through Fenton-type reaction process, Fe^2+^ may reduce hydrogen peroxide creating hydroxyl radicals which attack DNA inducing base lesions and single-strand breaks which may lead to double-strand breaks in return. ROS can also attack various important cellular proteins that are essential for DNA repair thus affecting their binding to the DNA substrates [16]. These cumulative effects of ROS which in most cases are endogenously generated by trace metal presence in the body cells are a key element of carcinogenic process. Even though the increase in oxidative DNA lesions has been frequently attributed to metal exposures, the molecular mechanism leading to tumor formation after such exposures is still not well understood.

## 2. Sampling and Sample Preparation Techniques

Proper sampling is very important to ensure accuracy and reliability of the results. Trace metal concentrations can be heterogeneously distributed in some tissues. As has been reported in many studies, the inhomogeneity of the trace metal concentrations depends on an individual's age, metal type, and tissue specimen under investigation [14]. The samples should be homogeneous as much as possible, and all handling and preparation devices and reagents must be checked carefully to avoid or reduce contamination as much as possible if reliable results are to be achieved. For effective PIXE analysis, the samples must be dry; however, the process must be carefully done to reduce tissue disturbance from its original physiological state. Much attention should be taken during sample extraction if cancerous and noncancerous tissues are examined since the distribution of malignant molecules is generally heterogeneous. For example, levels of Fe, Zn, and K in healthy breast tissues extracted near tumor affected areas are found to be higher than those obtained from healthy breasts tissues [4]. This has been attributed to the physiological process leading to the accumulation of trace elements in tumors, and increased enzymatic activities may have affected the composition of healthy tissue in the margin surrounding lesions [17]. Additionally, the increased levels of trace elements in cancerous tissues could be due to fibroglandular specimens as compared to noncancerous breast tissues.

The samples are collected by autopsy or by punch biopsy from normal and cancerous sites of deidentified patients. The samples are then quickly frozen in 2-methylbutane cooled in liquid nitrogen (−176°C). Rapid freezing reduces ice crystal formation and minimizes morphological damage to the samples. Frozen sections may be used for a variety of procedures, including immunochemistry, enzymatic detection, and in situ hybridization. Cryosectioning is a critical step as temperature gradients and sample freezing out processes may occur when sample is positioned in mounting medium that can facilitate cell disruption and ion displacement. During sectioning frozen tissue, the sample must be firmly attached to the microtome stage or chuck. Saline or water will hold the sample when frozen; however, embedding media are preferred since they provide a supportive and protective aid to sectioning. The most commonly used embedding medium is OCT, an aqueous solution of glycols and resins which provides an inert matrix for sectioning. 30% bovine albumin and von Apathy's gum syrup have been used as alternative to OCT. Once the frozen tissue is firmly in place in the cryostat (−40°C), the microtome lock is released and advanced or retracted to the chuck position until the knife edge just touches the block. The superficial surface of the sample is roughly trimmed in small steps (15–25 *μ*m) until an even, full face is achieved. The tissue debris is removed from the knife with a soft brush or tissue. Once the right cutting thickness is set and the cryostat and/or specimen allowed reaching the optimum cutting temperature, sections are cut using a slow even motion, except for hard tissue which requires a firmer stroke. The section should glide smoothly under the antiroll plate. Cutting is best done using a firm, steady motion and gentle pressure. Sections of 10–14 *μ*m thickness are cut and removed from the knife with a saline moistened brush. Alternatively, with unfixed tissue and using a cooled dry knife, the section may be picked up directly onto a glass slide where they will thaw on contact and adhere to the slide surface.

The sections are then freeze-dried. The freeze-drying process is used to remove water from the frozen tissue by sublimation of the solid phase at a low temperature and under vacuum. Sublimation can only occur when the partial pressure of the water vapor of the ice exceeds that of the atmosphere. In practice, the process of drying a frozen sample takes place under a vacuum of 10^−3^ Torr or greater and at a temperature difference sufficient to heat the ice crystals in the sample and provide energy for sublimation to water vapor. The transfer of water molecules from ice to vapor, however, removes heat from the environment causing a drop in temperature and a reduction in the rate of sublimation. A constant temperature must therefore be maintained in the sample so that sublimation proceeds at a rate equivalent to the heat input (−30 to −40°C). The samples are allowed to freeze-dry to about 1-2 *μ*m range and then mounted onto sample holders ready for irradiation. The sections can also be mounted on a foil of polycarbonate or silicon nitride adequately thin to enable high-resolution imaging using transmitted ions (STIM).

Other sample preparation techniques used in some of the studies involve freeze-drying the collected tissues and then converting into fine powder by pounding in an agate mortar. A fraction of this powdered material is dried overnight and mixed with a known quantity of internal standard like yttrium, and this mixture is again dried overnight. A known quantity of this powder is pressed into pellets, and these are used as targets.

## 3. PIXE as Analytical Technique

**Figure 1:**
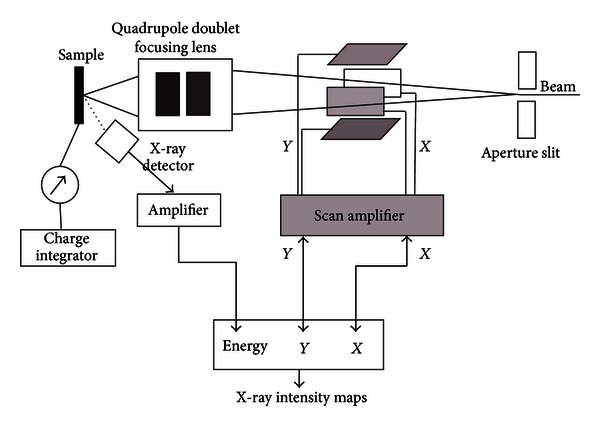
Schematic presentation of beam scanning and focusing for PIXE measurements in a typical microprobe setup.

In the recent years, most scientists have adopted PIXE as an analytical method of choice for trace elemental analysis in biological samples. This is due to its ability to produce more reliable results that can consistently be compared to other studies. PIXE is a noninvasive analytical technique that offers the most detection limits of parts per million (ppm) levels of sensitivity. This opens up new fields in biological microanalysis where the measurement of important trace elements such as calcium, iron, zinc, copper, and selenium among others now becomes possible on a microscale level. It is not an overstatement that microbeam PIXE is the only technique that offers ppm elemental sensitivities with high quantitative accuracy at spatial resolutions smaller than cell dimensions [18, 19]. The measurement of trace elements in individual cells is a field that is open to nuclear microscopy scientists and is expected to continue to grow significantly especially with the spatial resolution of nuclear microprobes reaching the 100 nm level as in most microprobe laboratories today. In PIXE, the interaction of MeV protons with a target leads to the ionization of the target atoms resulting into emission of X-rays with energies that provide characteristic spectral signatures of the target atoms. The well-characterized nature of these interactions alongside the ability to determine the proton energy loss and X-ray absorption within the target and the detector makes it possible to directly model X-ray production and calculate the X-ray yields for a given target composition. The accurate charge measurement using a charge integrator and possible secondary electron suppressor are necessary to ensure accuracy of the PIXE results. The suitability of PIXE method is further enhanced by the high cross-sections for PIXE X-ray production and low levels of continuum background [20–22].  Figure 1 shows the schematic presentation of beam scanning and focusing for PIXE measurements in a typical microprobe.

## 4. Trace Metal Concentrations in Human Tissues

**Figure 2:**
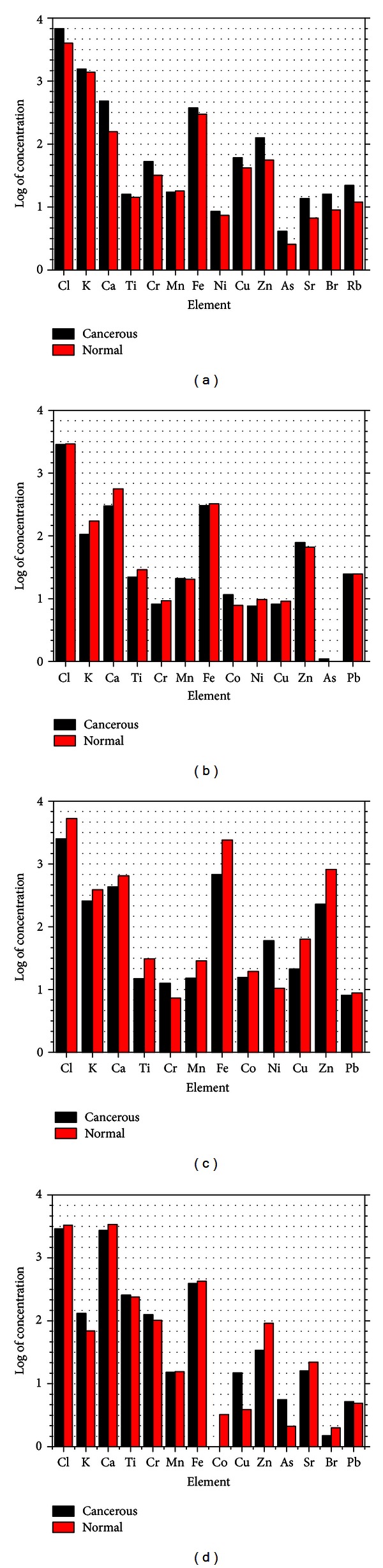
(a), (b), (c), and (d) show the bar charts of the log of concentrations of the trace elements for the breast, kidney, stomach, and testis tissues, respectively, which shows the correlation of the trace metal concentration of the cancerous and normal tissues of the selected organs.

There have been a lot of studies and large number of data on the levels of trace elements in human cancerous and noncancerous tissues obtained using different analytical techniques as mentioned earlier. Due to their vital role in metabolism of trace metals, liver, lung, and kidney have been more frequently studied. With the rise of other types of cancer, more recently, much interest has been laid on the study of other organs including the breast, prostate, penis, thyroid, and stomach. We will concentrate on the studies done using PIXE as analytical techniques. Data from the existing literature are reported in Table 1. Figures 2(a), 2(b), 2(c), and 2(d) show the bar charts of the log of concentrations of the trace elements for the breast, kidney, stomach, and testis tissues, respectively, which shows the correlation of the trace metal concentration of the cancerous and normal tissues of the selected organs.

### 4.1. Breast Tissue

Breast tissues were the most commonly studied sample compared to the others. One study found that the concentrations of the elements Cl, Ca, Cr, Fe, Cu, Zn, As, Se, BR, and Sr were significantly higher in cancerous tissues than normal tissues and that the concentrations of the elements K, Ti, and Ni had no significant difference from those of normal tissues [24]. These elevated levels were found to be similar to other previous studies that used other analytical techniques [17, 25–27]. The same study reported that the elevated level of chromium showed that it is a carcinogen as has been established. Chromium toxicity is commonly associated with exposure to hexavalent chromium compounds rather than to the low toxic trivalent chromium compounds [24]. Chromium (VI) has a characteristic ease of absorption by the body cells, and once in the cell, it is reduced to the trivalent state which produces genotoxic effects [28]. Its exposure can cause abnormal phenotypes due to generation of ROS and other several DNA lesions which in effect lead to DNA damage. Similarly, chromium has been found to repress p53 protein which is a tumor suppressor protein. Several studies reported that the inactivation of this protein is associated with various types of human cancers. P53 is involved in various biological processes including regulation of genes involved in the cell cycle, cell growth arrest after DNA damage, and apoptosis [29]. Due to the significance of cell cycle arrest and apoptosis, which are regulated by p53, their inactivation or alteration by reactive chromium intermediates such as Cr(V) and Cr(VI) can enhance cancer development [30]. Other carcinogen trace metals also include iron and copper. Fe catalyses hydrogen peroxide conversion to free radical ions which attack cellular membranes causing DNA strand breaks, inactivating enzymes, depolymerizing polysaccharides, and also initiating lipid peroxidation. Several studies have also established that Fe promotes inflammation and increases cancer cell growth [31, 32].

Due to its ability to change between its two oxidation statuses Cu^+^ and Cu^2+^, Cu has been found to cause generation of ROS which produce hydroxyl radicals that modify proteins, lipids, and nucleic acids eventually causing DNA damage [33]. Similarly, copper ions can aid carcinogenesis due to their role in angiogenesis which is a vital process in tumor progression [24].

Another study using the blood serum from breast cancer patients reported significant reduction in concentrations of Ti, Cr, Mn, Ni, Zn, and Se in the sera of breast cancer patients, which might be caused by cancer [34]. Previously, other studies have shown that there is a correlation between the increase in tissue levels of certain trace elements and their consequent decrease in the serum of breast cancer patients [24]. Studies have suggested that the ratio of Cu and Zn can be used for diagnostic or prognostic value in cancer. Similarly, the ratio of concentration of one metal in a tumor tissue to that of the same metal in nontumor tissue can be used for cancer diagnosis. For instance, one study found that the ratio of mean tumor to mean healthy concentration for Fe was 1.6 for paired samples and 2.7 for nonpaired samples, while that for Cu was found to be 3.1 and 3.6 for paired sample and nonpaired sample, respectively [17, 35].

### 4.2. Thyroid

Thyroid tissues have also been widely studied for trace metal. One study observed higher levels of Ti, V, Cr, Mn, Co, Fe, and Sr in cancerous tissues compared to the normal thyroid tissues [36]. While comparing their observation with another study which also studied trace elements in thyroid, both studies established consistent concentrations of K, Ca, and I, while variations were observed in the concentration of Cu, Zn, and Fe in cancerous thyroid compared to noncancerous tissues [36, 37]. Zn concentration in adenoma and cancerous thyroid was found to be lower than that in normal tissues, an observation consistent with other findings in the study of kidney and hair samples, respectively [38, 39]. Zn deficiency has been linked to severe deficiency in immune function and disruption in T-Cell function which is directly related to pathogenic carcinoma. Zn deficiency also causes inactivation of p53, a tumor suppressor protein, which has been associated with many cancers, including thyroid. Since iodine plays an important role in the functioning of thyroid glands, through the production of thyroid hormones (thyroxin and triiodothyronine) which are essential for cellular oxidation, growth, reproduction, and the activity of the central and autonomic nervous system, its low level can be attributed to cancer as observed by Reddy and his group in thyroid carcinoma tissues [36]. The malignant tumor tissues contain areas of necrosis and some hemorrhage caused by capillary ruptures. The rupture produces accumulation of hemoglobin from red blood cells which fragment into Fe containing heme, a process that leads to observed increased level of Fe in carcinoma tissues.

### 4.3. Kidney

Few studies have been done on kidney tissues. One such sturdy reported higher levels of Ti, Co, Zn, and Cd, and lower levels of K, Ca, Fe, Ni, and Se, respectively, in carcinoma kidney tissues compared to normal tissues [40]. The same study also observed that there was no variation of Cl, Cr, Mn, Cu, Br, and Pb between carcinoma tissues and the normal tissues. A different study, however, reported lower concentration of Cd, Cr, Ti, V, Cu, and Zn in cancerous kidney tissue than the noncancerous in the analysis, a fact this study attributed to the different metabolism and dynamics of cancer process compared to normal tissue [41]. Potassium being a major intracellular cation was observed to be in low concentration in carcinoma tissue than in normal tissue. It is known that prolonged potassium deficiency (known as hypokalemia) can cause injury to the kidney.

### 4.4. Stomach

With increasing cases of stomach cancer being reported, much attention has been turned to the study of stomach cancers. One such study reported higher concentrations of Cr, Ni, As, and Br and lower concentrations of Cl, K, Ca, Ti, Mn, Fe, Co, Cu, and Zn in stomach cancer tissues compared to noncancer tissues, respectively [40]. The high concentration of Cr in stomach cancer tissues supports its carcinogenic property [42]. Chromate ion enters the cells by sulfate uptake and is then reduced to Cr(III) through Cr(V)-glutathione intermediate species which binds with DNA producing a kinetically inert and damaging lesion. The same study observed that the low level of Fe in the stomach cancer tissue could be due to lack of HCl which may have resulted from the carcinoma nature of the stomach. Ni concentration was also much higher in the stomach cancer tissue, supporting its carcinogenic property as was reported in other studies [43, 44].

### 4.5. Penis and Testis

High concentration of Cu, Zn, and As and low concentration of Cl, Fe, and Co in the carcinoma tissue of penis compared to the normal tissue, respectively, have been reported [45]. The same study also reported high concentrations of K, Cr, and Cu and low concentrations of Cl, Ca, Ti, and Mn in carcinoma tissue of testis compared to normal tissue, respectively. The observed high concentration of Cu in the carcinoma tissue of both organs underlines the important role Cu plays in cancer promotion through inflammation and angiogenesis. A clinical study demonstrated that cancer can be fought by reduction of body copper levels, since it is the common denominator of angiogenesis [45]. They observed that cancer cells are easily proliferated into tumors in a high copper concentration environment, and thus most cancers growths can be inhibited through copper deficiency.

The Zn concentration levels were higher in the tissue of penis which supports the observations made in earlier works indicating that Zn is carcinogenic and that it is involved in tumor growth and development of neoplastic transformation [27, 28]. Zn has been found to enhance the activity of telomerase enzyme as well as antagonizing the inhibitory effect of bisphosphonates on breast and prostate tumor cell invasion as well [23]. In their study, Reddy and his group reported low concentration of Zn in testicular cancer and high Zn levels in penis cancer, an observation that suggests different roles played by Zn in different cancerous organs [40].

## 5. Discussion

The results shown in Table 1 demonstrate that there are differences and variations in trace metal concentrations from one organ to the other depending on their cancerous or noncancerous states. However, what is clear is that PIXE analysis can be effectively used to analyze trace metal concentrations of cancerous and noncancerous tissues. The variations of the trace metals can be attributed to the analysis technique used, sample sampling, and preparation technique including sample condition (wet/dry) and the reliability and accuracy level of the analytical technique. It is important to note that the PIXE technique would be very challenging for wet samples, while on the other hand drying the sample may also affect its physiology and hence the eventual results.

As analyzed for different organs above, the role of individual trace metals varies from one organ to the other. For example, one study reported low concentration of Zn in testicular cancer and high Zn levels in penis cancer [40]. Whether low or high, each different trace metal has specific effect on the carcinogenicity on the affected organ. The deficiency or excess of certain trace elements is correlated to the carcinogenesis of that organ. Other trace metals also act as inhibitors of cancer in different organs. For example, low levels of Se in the carcinoma tissue of kidney and low levels of Mn in carcinoma tissue of stomach, which supported the claims that they inhibit cancer growth in kidney and stomach, respectively, were reported by one group [36].

PIXE technique has this far proven to be a very sensitive technique for trace metal analysis especially of biological samples. When sampling and handling of samples during preparation are properly done, this analytical technique promises to be one of the best since a wide range of trace metals can easily be qualitatively and quantitatively detected. The use of a more sensitive detector like HPGe-detector can also improve the analysis especially of low-energy X-rays from lighter elements.

## 6. Conclusion

From the observations made in this paper, PIXE analysis can be successfully used to determine trace elemental concentrations of cancerous and noncancerous human tissues. The low or high levels of the trace metals in cancerous or noncancerous tissues can be used to determine the carcinogenic role of the deficiency or excess of each trace metal in specific organs. These concentration levels need to be closely monitored in further prognostic studies. The results also need to be verified by other advanced diagnostic techniques like high throughput sequencing, gene expression sequencing, and proteomics for conclusive determination to be made. For result reliability and correct assessment of the role of each trace metal in regard to carcinogenesis, the role in initiation, promotion, progression, or inhibition of cancer in various organs, there is need for acquisition of more data from several investigations. Similarly, there is a need to carry out the investigations from different regions using differentials like gender and age dietary habits among other variables.

## Figures and Tables

**Table 1:** Trace metal levels reported in the literature for cancerous and noncancerous human tissues using PIXE. The values are in *μ*g/g.

Tissue	Tissue type	Cl	K	Ca	Ti	V	Cr	Mn	Fe	Co	Ni	Cu	Zn	As	Sr	I	Hg	Se	Br	Rb	Pb	Reference
Breast	Normal	3999	1381	157	14.3	—	31.9	18	299	—	7.4	42	56	2.57	6.7	—	—	0.66	9	12	—	[24]
	Cancerous	6815	1550	480	15.9	—	52.7	17.2	376	—	8.56	60.7	126	4.12	13.7	—	—	1.32	16	22	—	

Breast (serum)	Normal	—	—	—	416	32	18.6	29.8	291	2.9	7.2	24.6	38.5	0.9	—	—	—	2.5	46	—	—	[34]
	Cancerous	—	—	—	238	19	10	16	355	0.9	5.3	32.3	13	0.69	—	—	—	1.5	23	—	—	

	Normal	200	11.6	688	18	4.5	6	17	569	11	8.4	54.9	149	125	11.7	229	98	—	—	—	17	
Thyroid	Adenoma	215	18	577	15.9	4.2	36.6	14.4	525	11.6	10.6	6	68.7	17	6.8	222	19.8	—	—	—	11.6	[36]
	Cancerous	234	159	393	103	20	29.8	46.6	1397	17.9	7.7	12.9	71.8	2.8	—	—	7.7	—	—	—	17.8	

Kidney	Normal	2911	172	562	22	—	9.3	20.4	325	7.8	9.7	9.1	66	—	—	—	—	1.8	9.8	—	24.7	[40]
	Cancerous	2857	106	298	29	—	8.2	20.9	305	11.6	7.6	8.2	78	1.1	—	—	—	—	8.2	—	24.6	

Stomach	Normal	5295	387	647	31	—	7.3	28.6	2408	19.4	10.5	63.5	818	—	—	—	—	—	—	—	8.8	[40]
	Cancerous	2518	256	433	15	—	12.6	15.1	684	15.6	60	21.2	229	1.71	—	—	—	—	3.1	—	8.1	

Penis	Normal	3583	38.3	877	15.2	—	4.6	10.3	650	25	—	7.5	58.2	2.1	22	—	—	—	2	—	4.9	[45]
	Carcinoma	2133	57.8	815	16.8	—	2.6	9.2	464	11	—	79.6	148	5.6	16	—	—	—	1.5	—	5.2	

Testis	Normal	3301	69	3400	238	—	102	15.6	426.4	3.25	—	3.9	91.7	—	—	—	—	—	—	—	—	[45]
	Carcinoma	2887	131	2726	257	—	125	15.2	391.9	0.9	—	14.9	34	—	—	—	—	—	—	—	—	
